# Sharing all types of clinical data and harmonizing journal standards

**DOI:** 10.1186/s12916-016-0612-8

**Published:** 2016-04-03

**Authors:** Corrado Barbui

**Affiliations:** WHO Collaborating Centre for Research and Training in Mental Health and Service Evaluation, Section of Psychiatry, University of Verona, Verona, Italy

**Keywords:** Accountability, Data sharing, Research methodology

## Abstract

Despite recent efforts to enforce policies requiring the sharing of data underlying clinical findings, current policies of biomedical journals remain largely heterogeneous. As this heterogeneity does not optimally serve the cause of data sharing, a first step towards better harmonization would be the requirement of a data sharing statement for all clinical studies and not simply for randomized studies. Although the publication of a data sharing statement does not imply that all data is made readily available, such a policy would swiftly implement a cultural change in the definition of scientific outputs. Currently, a scientific output only corresponds to a study report published in a medical journal, while in the near future it might consist of all materials described in the manuscript, including all relevant raw data. When such a cultural shift has been achieved, the logical conclusion would be for biomedical journals to require authors to make all data fully available without restriction as a condition for publication.

## Background

The recently released US Institute of Medicine (IOM) report [1] on clinical data sharing has stimulated a wide-ranging discussion on the benefits and harms associated with the unrestricted availability of all data underlying clinical findings [2, 3]. The IOM concluded that the benefits of sharing outweigh the risks, and made suggestions for further improvement. Further, the report urged biomedical journals, as evaluators and publishers of research results and implementers of academic standards, to enforce policies that require the sharing of clinical trial data [1]. In response to this, in 2016, the International Committee of Medical Journal Editors (ICMJE) proposed the requirement that de-identified individual patient data underlying the results presented in a given article should be provided as a condition for consideration of publication of a clinical trial report [4].

As a practical contribution to this challenging debate, the present commentary aims to raise awareness on the current data sharing policies of biomedical journals. As an example, the instructions for authors of the top ten general and internal medical journals according to impact factor were inspected, and information on data sharing requirements were abstracted and summarized.

## Current data sharing policies of biomedical journals

The top ten general and internal medicine journals according to impact factor currently implement three different data sharing approaches (Table 1). A first group of journals, including the *New England Journal of Medicine*, *The Lancet*, *JAMA*, *JAMA Internal Medicine*, *Journal of Cachexia and Sarcopenia*, and *Mayo Clinic Proceedings*, do not mention data sharing, do not require any statement to be published along with the study report on the possibility to access the raw data, and do not suggest repositories or other tools to make the raw data available. A second group of journals, including *Annals of Internal Medicine*, *BMJ*, and *BMC Medicine*, encourage data sharing and require a formal statement describing the conditions under which raw data are accessible. Additionally, from July 1, 2015, the *BMJ* requires that all submitted clinical trials include a declaration of data sharing upon request [5]. Finally, a third policy is implemented by *PLoS Medicine*, requiring full availability of all data underlying the findings described in published study reports (Table 1). In general, journals recommending or requiring data sharing also suggest repositories and other tools to make the raw data available.

**Table 1:** Data sharing requirements of the top ten general and internal medicine journals

Journal	Data sharing			Data sharing statement	Data repositories suggested
	Not mentioned	Suggested	Required		
New England Journal of Medicine	X			No	No
The Lancet	X^a^			No	No
JAMA	X			No	No
Annals of Internal Medicine		X^b^		Yes	No
BMJ		X^c^		Yes	Yes
PLoS Medicine			X^d^	Yes	Yes
JAMA Internal Medicine	X			No	No
Journal of Cachexia and Sarcopenia	X			No	No
BMC Medicine		X^e^		Yes	Yes
Mayo Clinic Proceedings	X			No	No

^a^ Authors may be required to provide the raw data for research papers when they are under review and up to 10 years after publication in *The Lancet*

^b^ *Annals of Internal Medicine* encourages but does not typically require the sharing of the raw data. However, it requires that authors state their willingness to share and any conditions for sharing

^c^ For all trials, the *BMJ* requires data sharing on request as a minimum

^d^ *PLoS Medicine* requires authors to make all data underlying the findings described in their manuscript fully available without restriction, with rare exception

^e^ *BMC Medicine* strongly encourages that all datasets on which the conclusions of the paper rely should be made available to readers.

## Towards a new sharing culture

Current diversity in journal policies does not optimally serve the cause of data sharing as it allows varying academic standards. Through this system, study authors are able to choose their standard of preference when submitting a study report – a decision which might be guided, among other considerations, by their willingness to share.

As a first step towards better harmonization, biomedical journals should require a data sharing statement for all types of clinical study reports, and not simply for randomized studies. If audit and accountability are the ‘bread and butter’ of good medicine and science [6], accepting various policies for different clinical study types would imply that studies with an observational design are not good science.

Even if publishing a data sharing statement does not mean making all data available, such a policy would swiftly implement a cultural change in the definition of scientific outputs. Currently, a scientific output only corresponds to a study report published in a medical journal, while in the near future it might consist of all materials described in the manuscript, including all relevant raw data.

With such a policy uniformly implemented, researches, who are currently interested in designing and conducting studies with the aim of meeting the highest methodological standards and requirements to ensure publication in major medical journals, would consider the issue of data sharing from the inception of their research projects. This would imply, for example, (1) the inclusion of a data sharing plan as part of a study protocol and its registration in international repositories of study protocols [7]; (2) agreement with local ethics committees on a procedure to preserve patient confidentiality and privacy when de-identified individual patient data are shared [8, 9]; (3) the inclusion of financial support for data sharing in grant applications; (4) drafting of a detailed publication plan in order to allow the best use of the database [10]; and, even more importantly, (5) the development of a high-quality database in a way suitable for secondary uses, written and coded in English, for example, but also meeting other requirements that expert methodologists would need to further develop and define [11]. There should also be careful development of web-based infrastructures for open data, as it would be rather disappointing if the promising development of open sharing of data led to no more than researchers piling their data in fairly unsearchable data repositories [12, 13]. Additionally, reporting guidelines, such as the CONSORT for clinical trials, PRISMA for systematic reviews of clinical trials, STROBE for observational studies, and MOOSE for systematic reviews of observational studies, would need to be updated by adding items for proper data sharing plans (what to share, when, and how) [7].

This policy would make data sharing the norm, with some reasonable exceptions that authors may publicly declare in their data sharing statement [14]. As the majority of published studies are not clinical trials, but rather studies with an observational design, it may be expected that most researchers would easily adhere to the spirit and practicalities of data sharing. Paradoxically, therefore, observational rather than randomized data may pave the way towards full implementation of a data sharing culture.

## Conclusions

When such a cultural shift has been achieved, the logical conclusion would be for biomedical journals to require authors to make all data fully available without restriction as a condition for publication. In 2004, the ICMJE announced that it would require registration of clinical trials as a condition for publication, with a remarkable effect in clinical trial research [15]. If the top-ranking biomedical journals, for example those belonging to the ICMJE, were to find a consensus on these steps and homogenize their standards on data sharing for all types of clinical studies, then the remaining journals would certainly follow.
